# An electrical stimulation intervention protocol to prevent disuse atrophy and muscle strength decline: an experimental study in rat

**DOI:** 10.1186/s12984-023-01208-6

**Published:** 2023-06-29

**Authors:** Haiwang Shi, Fan Li, Fulong Zhang, Xiaobei Wei, Chengyi Liu, Rui Duan

**Affiliations:** Lab of Regenerative Medicine in Sports Science, School of Physical Education and Sports Science, South China Normal University, Guangzhou, China

**Keywords:** CSA, Disuse Atrophy, Fiber type, MES, Muscle strength

## Abstract

**Background:**

Skeletal muscle is negatively impacted by conditions such as spaceflight or prolonged bed rest, resulting in a dramatic decline in muscle mass, maximum contractile force, and muscular endurance. Electrical stimulation (ES) is an essential tool in neurophysiotherapy and an effective means of preventing skeletal muscle atrophy and dysfunction. Historically, ES treatment protocols have used either low or high frequency electrical stimulation (LFES/HFES). However, our study tests the use of a combination of different frequencies in a single electrical stimulation intervention in order to determine a more effective protocol for improving both skeletal muscle strength and endurance.

**Methods:**

An adult male SD rat model of muscle atrophy was established through 4 weeks of tail suspension (TS). To investigate the effects of different frequency combinations, the experimental animals were treated with low (20 Hz) or high (100 Hz) frequency before TS for 6 weeks, and during TS for 4weeks. The maximum contraction force and fatigue resistance of skeletal muscle were then assessed before the animals were sacrificed. The muscle mass, fiber cross-sectional area (CSA), fiber type and related protein expression were examined and analyzed to gain insights into the mechanisms by which the ES intervention protocol used in this study regulates muscle strength and endurance.

**Results:**

After 4 weeks of unloading, the soleus muscle mass and fiber CSA decreased by 39% and 58% respectively, while the number of glycolytic muscle fibers increased by 21%. The gastrocnemius muscle fibers showed a 51% decrease in CSA, with a 44% decrease in single contractility and a 39% decrease in fatigue resistance. The number of glycolytic muscle fibers in the gastrocnemius also increased by 29%. However, the application of HFES either prior to or during unloading showed an improvement in muscle mass, fiber CSA, and oxidative muscle fibers. In the pre-unloading group, the soleus muscle mass increased by 62%, while the number of oxidative muscle fibers increased by 18%. In the during unloading group, the soleus muscle mass increased by 29% and the number of oxidative muscle fibers increased by 15%. In the gastrocnemius, the pre-unloading group showed a 38% increase in single contractile force and a 19% increase in fatigue resistance, while in the during unloading group, a 21% increase in single contractile force and a 29% increase in fatigue resistance was observed, along with a 37% and 26% increase in the number of oxidative muscle fibers, respectively. The combination of HFES before unloading and LFES during unloading resulted in a significant elevation of the soleus mass by 49% and CSA by 90%, with a 40% increase in the number of oxidative muscle fibers in the gastrocnemius. This combination also resulted in a 66% increase in single contractility and a 38% increase in fatigue resistance.

**Conclusion:**

Our results indicated that using HFES before unloading can reduce the harmful effects of muscle unloading on the soleus and gastrocnemius muscles. Furthermore, we found that combining HFES before unloading with LFES during unloading was more effective in preventing muscle atrophy in the soleus and preserving the contractile function of the gastrocnemius muscle.

## Background

Skeletal muscle atrophy is commonly associated with aging, malnutrition, fasting and disuse conditions such as bed rest, inactivity, and microgravity [1, 2], resulting in a decrease in muscle mass and fiber cross-sectional area (CSA). Muscle atrophy is also frequently accompanied by changes in muscle fiber type and declining contractile function [3–5], leading to impaired mobility and inconvenience [6], increased risk of injury, and slower recovery [7, 8]. The imbalance between skeletal muscle protein synthesis and degradation, and the shift in muscle fiber types are the main causes of skeletal muscle atrophy [9–13]. Exercise is a proven and effective method of treating skeletal muscle atrophy by promoting protein synthesis through the PI3K/AKT/mTOR pathway and reducing protein degradation through the downregulation of MuRF1 expression [14–18]. However, exercise may not be feasible for individuals with trauma or post-surgery, making alternative methods of muscle contraction necessary.

In the 1960s, the field of physical medicine introduced physiological electrical stimulation (ES), a technique that simulates muscle contraction. This led to the development of a functional electrical stimulation system for clinical use, designed to trigger useful muscle contraction and maintain muscle quality and function [19, 20]. Today, ES is not only used as a rehabilitation treatment [21–23], but also as an effective training tool for people with physical weakness and for the general population to improve their sports performance [24]. Studies have shown that different electrical stimulation frequencies have varying effects on skeletal muscles. High frequency electrical stimulation (HFES, > 40 Hz in general) mimics resistance training to promote skeletal muscle protein synthesis and increase muscle mass, helping to prevent the decline of muscle mass and contractility that often occurs with aging and denervation [25–27]. Low frequency electrical stimulation (LFES, 5 to 30 Hz in general) mimics aerobic exercise and promotes the biological activity of mitochondria [23, 28–31]. Noteworthy, frequencies under 16 Hz were not strong enough to produce a significant contraction [32]. Although the contraction force generated by LFES (10-30 Hz) is low, the duration of the contraction force can last 24 h or longer, an effect not seen with higher frequency stimulation [19]. Despite the extensive research on the effects of single-frequency electrical stimulation on skeletal muscles, there have been relatively fewer studies exploring the impact of combining high and low frequency electrical stimulation on skeletal muscle mass and contractile function. This remains an area of active research, as researchers aim to better integrate electrical stimulation protocols with specific treatment goals and develop effective stimulation treatment plans to improve the contractility and fatigue resistance of skeletal muscles.

In this study, various treatment regiments combining high frequency or low frequency electrical stimulation at different stages of muscle atrophy were used to evaluate their effects. The maximum contractile and fatigue resistance of skeletal muscle, skeletal muscle weight, fiber CSA were measured to determine the most effective treatment regimen. Our results showed that a pretreatment with HFES can mitigate the negative impact of muscle unloading on the soleus and gastrocnemius muscles. Additionally, LFES treatment during unloading, following HFES pretreatment, was found to be more effective in resisting soleus muscle atrophy and preventing a decline in the contractile function of gastrocnemius muscle. The protein expression of nuclear factor of activated T cell (NFAT) and calcium/calmodulin-dependent phosphatase calcineurin (CaN), which can impact the fiber type of skeletal muscle [33, 34], were also detected using Western Blot analysis.

## Materials and methods

### Animals

**Fig. 1:**
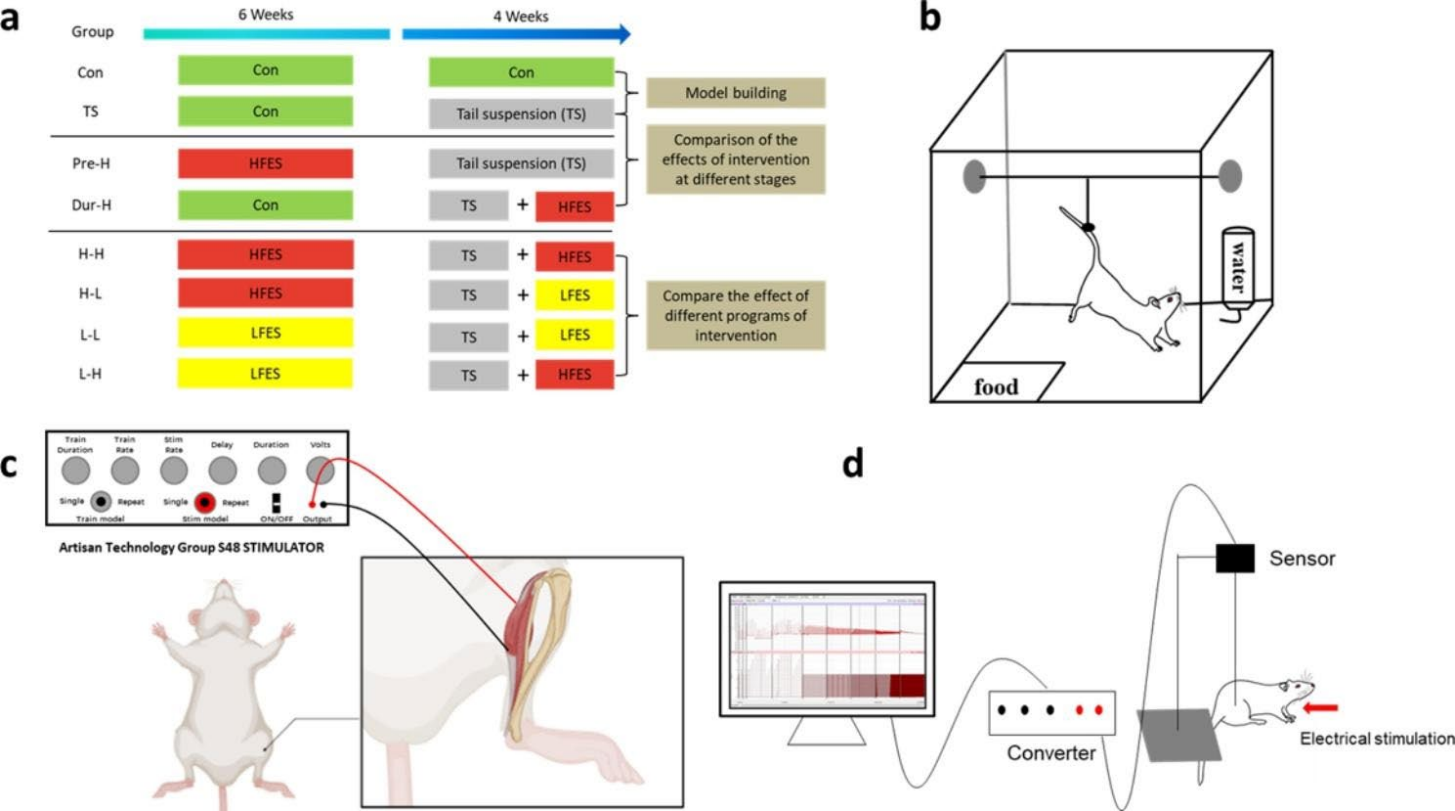
**The experimental method** (**a**). Research group. (Con: control group, TS: Tail suspension group, Pre-H: HFES before TS intervention, Dur-H: HFES during TS, H-H: HFES before and during TS, H-L: HFES before TS plus LFES during TS, L-H: LFES before TS plus HFES during TS, L-L: LFES before and during TS). (**b**). Skeletal muscle atrophy model: rat tail suspension. (**c**). Illustration of Electrical stimulation on muscle. In the electrical stimulation procedure, two steel needles were used as electrodes and connected to the stimulator. The positive electrode was positioned behind the junction of the two gastrocnemius muscle bundles, near the peroneal nerve, while the negative electrode was placed at the lower end of the ventral part of the gastrocnemius muscle and close to the Achilles tendon. (**d**). To test the mechanical properties of the gastrocnemius muscle, a wire was attached to the distal end of the gastrocnemius tendon. External electrical stimulation was applied, causing the gastrocnemius muscle to contract, and allowing the measurement of the mechanical changes resulting from different electrical stimulation signals

In this study, 43 adult male Sprague-Dawley rats (aged 10 weeks) were randomly divided into eight groups, as depicted in Fig. 1a. The groups were: control (Con, n = 6), tail suspension (TS, n = 6), HFES before TS intervention (Pre-H, n = 5), HFES during TS (Dur-H, n = 6), HFES before and during TS (H-H, n = 5), LFES before and during TS (L-L, n = 5), HFES before TS plus LFES during TS (H-L, n = 6) and LFES before TS plus HFES during TS (L-H, n = 4). The rats were housed in individually ventilated cages under standard housing conditions (22 °C, 12 h light/dark cycle), with unlimited access to food and water. Chloral hydrate was used to anesthetize the animals during ES treatment. Animal procedures were approved by Animal Care and Use Committee of South China Normal University.

### Tail suspension

The rat tail suspension (TS) procedure is commonly used method to induce muscle atrophy in rats. In brief, the rat’s tail was secured with pressure-sensitive tape and gauze, and the body was suspended at a 30° angle to the horizontal surface with the forelimbs still able to touch the floor of the cage and access to food and water [35, 36] (as shown in Fig. 1b). The tail adhesion position was checked after each electrical stimulation treatment to avoid falling or injury. All groups, except the control group, underwent tail suspension for 4 weeks.

### Electrical stimulation program

The Artisan Technology Group S48 STIMULATOR was employed to deliver electrical stimulation to the gastrocnemius and soleus muscles in rats. Two sterile stainless-steel needles, with a diameter of 0.3 mm and length of 13 mm, were connected to the cathode and anode of the stimulator and pulses were transmitted between the needles to cause skeletal muscle contractions. To specify the exact electrode placement, the cathode (negatively charged electrode) was positioned 5 mm below the fibulae head, laterally and posteriorly to the knee-joint, in close proximity to the superficial fibular nerve and deep fibular nerve. On the other hand, the anode (positive electrode) was located externally and inferiorly to the caput fibulae, near the tibial nerve [37]. The depth of needle insertion varied from 0.5 to 1.0 cm, depending on the thickness of skin and fatty tissue. The stimulation protocol aimed to recruit hindlimb gastrocnemius and soleus muscle contractions (as shown in Fig. 1c). To minimize the impact of other factors, the Con and TS group received the same anesthesia and acupuncture procedure as the ES treatment group, but without electrical stimulation (SHAM treatment). All experimental animals received ES or SHAM treatment for 6 weeks before and 4 weeks during TS. ES treatment was administered every two days and the stimulation parameters are presented in Table 1 [23, 25, 38, 39].

**Table 1:** Electrical Stimulation Scheme

Scheme	Fre/Hz	Volt/V	Duration/ms	Delay/ms	Stimulation time	Interval time
HFES	100	10	1	10	3 s	7 s
LFES	20	8	9	90	45 min	-

HFES treatment consists of 5 sets of stimulation, with each set consisting of 10 repetitions of 3 s of stimulation followed by 7 s of interval. The interval between each set was 10 min, for a total of 45 min of stimulation; LFES treatment consists of a total of 45 min of continuous stimulation.

### Gastrocnemius mechanical performance test

To assess the impact of unloading and electrical stimulation on skeletal muscle function, the single contractile force and fatigue resistance were measured using the I-WORX muscle strength test system (I-Worx RA 834, USA). The evaluators responsible for conducting the measurements were blinded to the treatment group assignments, ensuring unbiased assessments of the muscle function. The force transducer’s output was recorded by an oscilloscope (546601B; Hewlett-Packard, Palo Alto, CA) and a chart recorder (BD-11E; Kipp and Zonen, Delft, The Netherlands). The Achilles tendon of the gastrocnemius muscle was sectioned and attached to a sensor (Fig. 1d). The gastrocnemius muscle was electrically stimulated 10 times with a frequency of 5 Hz and voltage of 10 V to measure its single contractile force [40]. The fatigue resistance of gastrocnemius muscle was determined through stimulation at frequencies of 0.25 Hz, 0.33 Hz, 0.4 Hz, 0.5 Hz, 1 Hz with a constant voltage of 5 V, applied 300 times. The gastrocnemius muscle’s fatigue resistance was calculated by dividing the muscle strength reduction value by the single contraction force after undergoing the fatigue protocol.

### Sample preparation and histological analysis

One day after the final electrical stimulation session, all rats were humanely euthanized with an overdose of isoflurane. The soleus and gastrocnemius muscle were then harvested, weighed, embedded in a tragacanth matrix, and frozen in liquid nitrogen-cooled isopentane for histochemical analysis.

### Histochemical

Transverse sections of 8-µm were prepared using a cryostat microtome (Leica CM 1850, Germany). These sections were then stained for myofiber adenosine triphosphatase (ATPase) following pre-incubation at pH 4.5 and 4.2, to distinguish between type I and type II muscle fibers, respectively [41, 42]. For hematoxylin and eosin (HE) staining, transverse 8-µm sections were taken from the midpoint of the muscle proximal-distal axis, fixed with 4% formaldehyde, and stained with hematoxylin and eosin. CSA and fiber type of 600–700 soleus muscle fibers and 1200–1400 gastrocnemius muscle fibers were analyzed through Image J software (NIH, Bethesda, MD, USA) for each rat. To ensure the results of the experiment are not biased, all evaluators in assessing the histology were blinded to the treatment group information.

### Western bloting

The tissues of the soleus muscles were extracted and lysed using RIPA buffer (Beyotime, China). The protein concentration was the determined using BCA method, followed by boiling and centrifugation. For SDS-PAGE analysis, 30 ug of protein was loaded and separated. The resulting protein was then transferred to PVDF membranes (Millipore, USA) and blocked with 5% non-fat dry milk for one hour at room temperature. After washing with PBST, the membranes were incubated with primary antibodies against NFATC2 (1:1000, CST, USA), CaN (1:1000, CST, USA), or GAPDH (1:500, Santa Cruz, USA) at 4 °C overnight, and subsequently incubated with goat anti-rabbit-HRP (1:2000, Beyotime, China) secondary antibodies at room temperature for 1 h. The presence of immunoreactive bands was detected using ChemiDoc™ Touch Imaging System (Bio-Rad, USA).

### Statistical analysis

Statistical analysis was performed using SPSS software (SPSS Inc., Chicago, IL) and results were presented as the mean ± standard deviation (SD). We have used the Bonferroni method to correct the P value, and then the Independent Samples T-test was used to compare the difference among groups during the post-hoc multiple comparison. Graphical representations of the results were generated using GraphPad Prism 8 software (GraphPad Software, San Diego, USA).

## Results

### Body weight and muscle mass

**Fig. 2:**
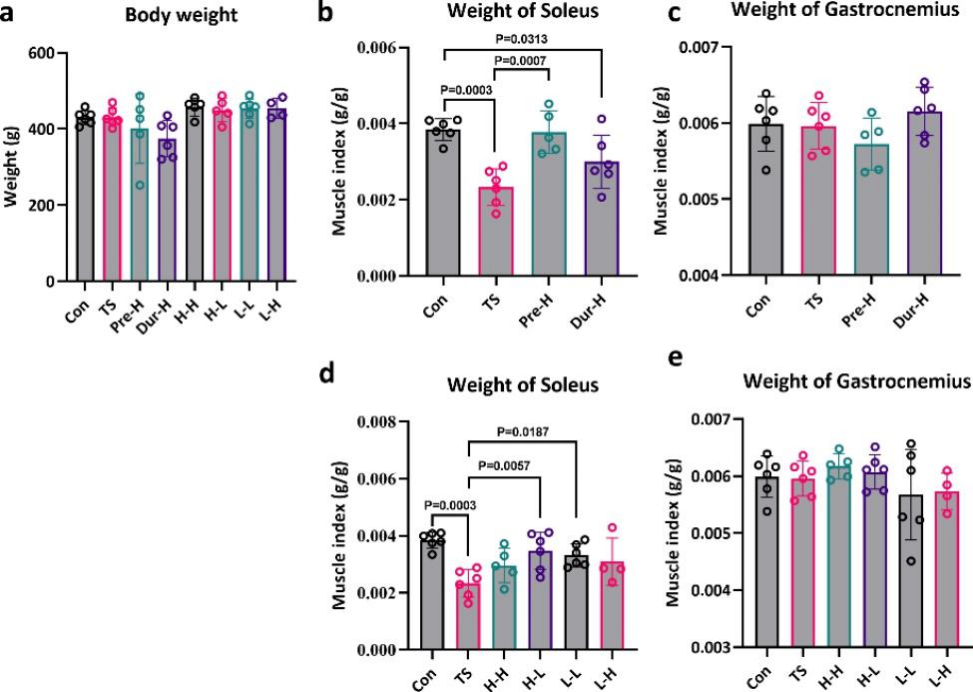
**The body weight of rats at the time of sacrifice and unloading causes skeletal muscle atrophy, and electrical stimulation therapy maintains skeletal muscle mass** (**a**). Body weight of rats at sacrifice. (**b**, **c**). The effect of different intervention stages on skeletal muscle quality. (**d**, **e**). The effect of different stimulation schemes on skeletal muscle quality (Con: control group, TS: Tail suspension group, Pre-H: HFES before TS intervention, Dur-H: HFES during TS, H-H: HFES before and during TS, H-L: HFES before TS plus LFES during TS, L-H: LFES before TS plus HFES during TS, L-L: LFES before and during TS)

Previous studies have shown that HFES is more effective in increasing muscle mass compared to LFES. We conducted an experiment to determine the effect of HFES before or with TS. After four weeks of TS, there was no change in body weight among the groups (Fig. 2a), but the soleus muscle mass was reduced by 39% (Fig. 2b). HFES administered before or with TS was found to alleviate the loss of soleus muscle mass (Fig. 2b). It is suggesting that skeletal muscle atrophy can not only be prevented during TS but also beforehand. Thus, we used a combination of low or high frequency electrical stimulation before or during TS to find the most effective method to combat skeletal muscle atrophy (Fig. 1a). Our findings showed that the H-L and L-L groups had a significant increase in soleus muscle weight compared with TS group, but there was no difference between H-L and L-L group (Fig. 2d). There was no difference in gastrocnemius muscle weight between any of the groups (Fig. 2c, e).

### Cross section area

**Fig. 3:**
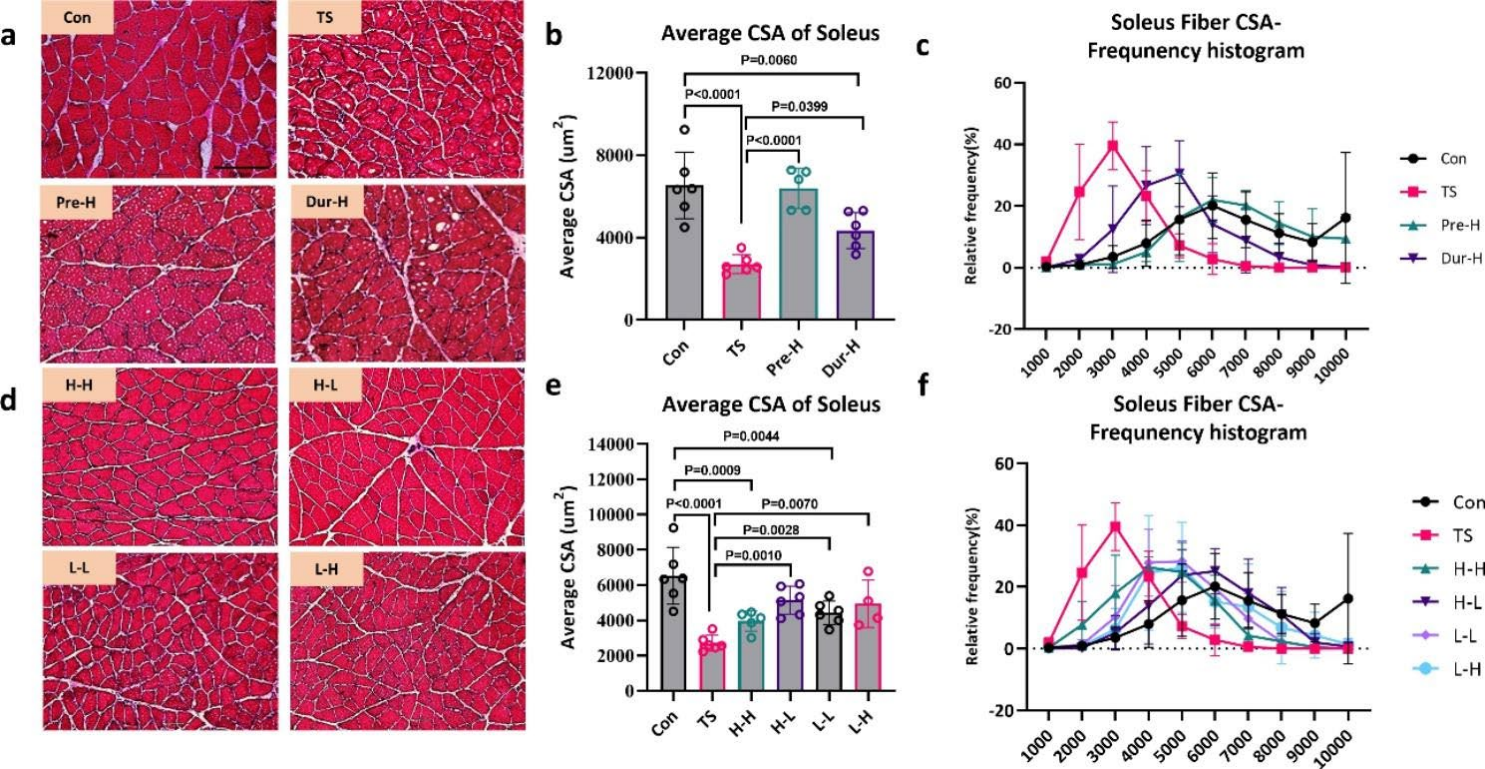
**Skeletal muscle electrical stimulation can resist the decrease in the CSA of soleus muscle fibers due to unloading** (**a**, **d**). The Hematoxylin eosin (HE) staining of soleus muscle, the scale bar represents 100 μm. (**b**, **e**). The influence of the CSA of soleus muscle fibers in different intervention stages and different electrical stimulation treatments. (**c**, **f**). The influence of frequency domain on the CSA distribution of soleus muscle fibers in different intervention stages and different electrical stimulation treatments (Con: control group, TS: Tail suspension group, Pre-H: HFES before TS intervention, Dur-H: HFES during TS, H-H: HFES before and during TS, H-L: HFES before TS plus LFES during TS, L-H: LFES before TS plus HFES during TS, L-L: LFES before and during TS)

**Fig. 4:**
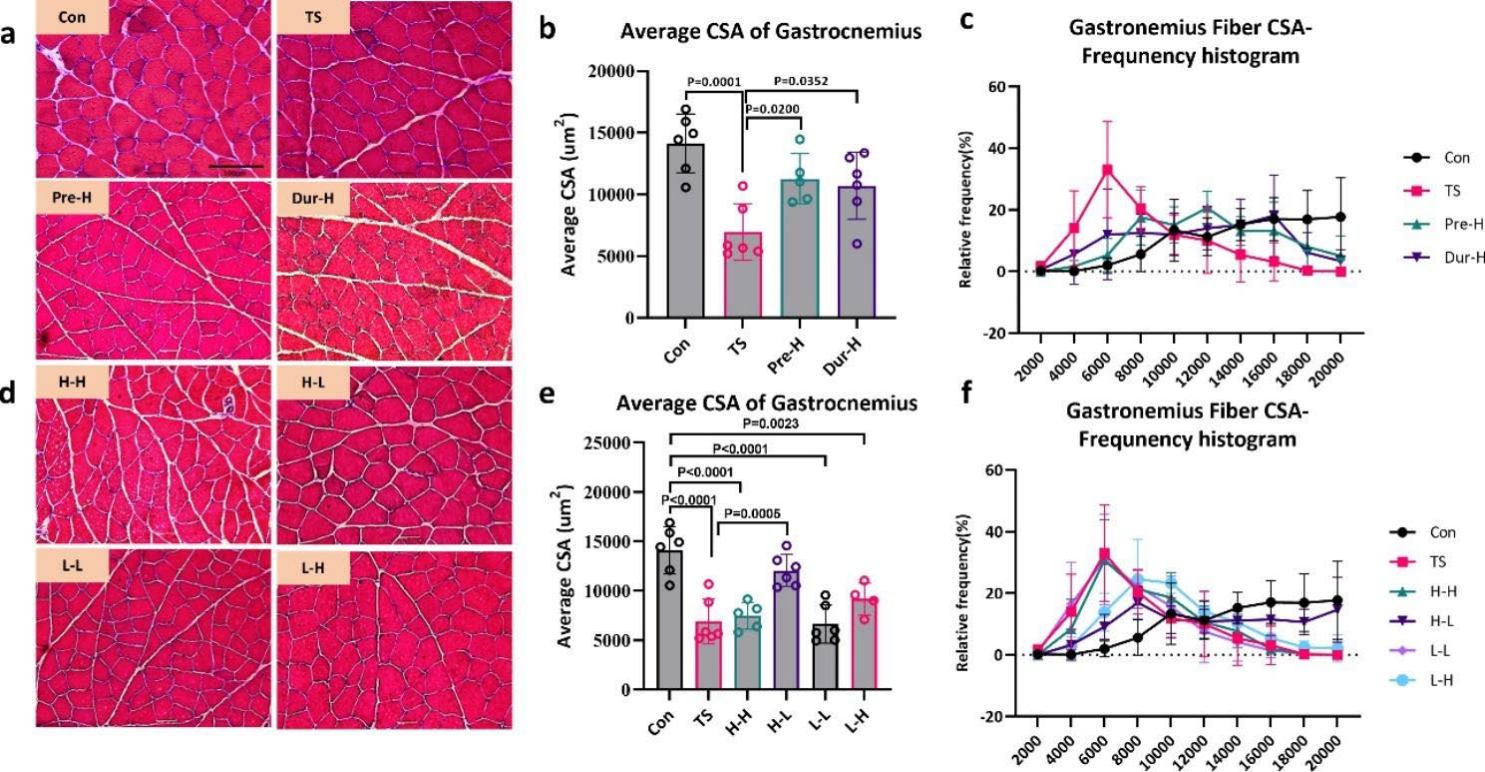
**Skeletal muscle electrical stimulation can resist the decrease in the CSA of gastrocnemius muscle fibers due to unloading** (**a**, **d**). The Hematoxylin eosin (HE) staining of gastrocnemius muscle, the scale bar represents 100 μm. (**b**, **e**). The influence of the CSA of gastrocnemius muscle fibers in different intervention stages and different electrical stimulation treatments. (**c**, **f**). The influence of frequency domain on the CSA distribution of gastrocnemius muscle fibers in different intervention stages and different electrical stimulation treatments (Con: control group, TS: Tail suspension group, Pre-H: HFES before TS intervention, Dur-H: HFES during TS, H-H: HFES before and during TS, H-L: HFES before TS plus LFES during TS, L-H: LFES before TS plus HFES during TS, L-L: LFES before and during TS)

After four weeks of unloading, a 58% decrease was observed in the CSA of soleus fibers and a 51% decrease in the CSA of gastrocnemius fibers (Fig. 3b). The CSA of fibers in soleus and gastrocnemius muscles in the control group was concentrated in the range of 2500–4000 µm^2^ but decreased to 1000–2000 µm^2^ after four weeks of unloading (Figs. 3c and 4c). Pre-training with HFES for six weeks before unloading prevented the decline in CSA in the soleus and gastrocnemius muscles induced by TS (Figs. 3c and 4c). On the other hand, HFES treatment during unloading only maintained gastrocnemius CSA (Fig. 4b) but not soleus CSA (Fig. 3b).

The results of CSA in the combined intervention group showed a similar trend as the changes in skeletal muscle mass. The H-L, L-H and L-L groups all alleviated the decline in CSA induced by unloading, and maintained CSA within the range of 2000 to 3000 µm^2^, except for H-H electrical stimulation group (Fig. 3e, f). Among these groups, the effect of H-L was the best, which was consistent with the muscle weight.

### Muscle single contractile force and fatigue resistance

**Fig. 5:**
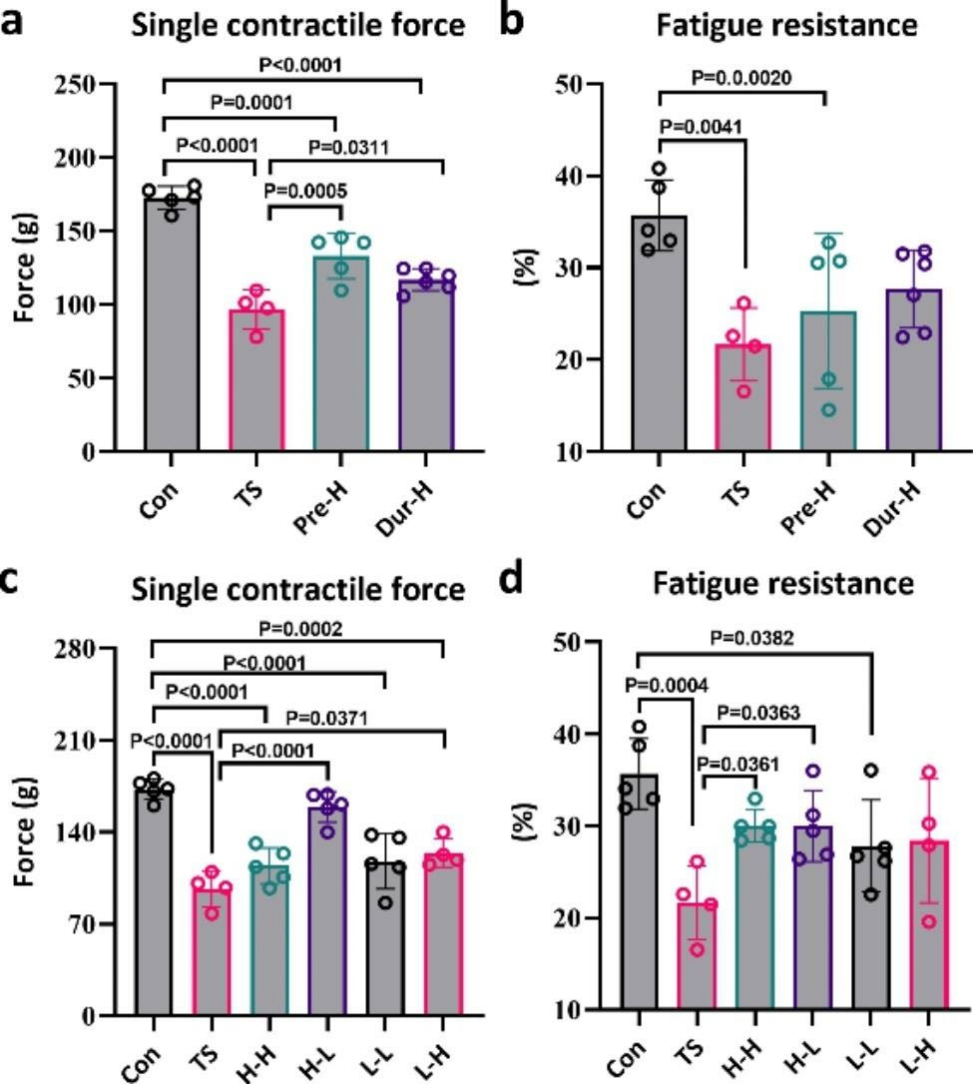
**Electrical stimulation can reverse the decrease in gastrocnemius contraction caused by unloading** (**a**). The effect of different intervention stages on single contraction force of gastrocnemius muscle. (**b**). The effect of different intervention stages on fatigue resistance of gastrocnemius muscle. (**c**). The effect of different electrical stimulation schemes on single contraction force of gastrocnemius muscle. (**d**). The effect of different electrical stimulation schemes on fatigue resistance of gastrocnemius muscle (Con: control group, TS: Tail suspension group, Pre-H: HFES before TS intervention, Dur-H: HFES during TS, H-H: HFES before and during TS, H-L: HFES before TS plus LFES during TS, L-H: LFES before TS plus HFES during TS, L-L: LFES before and during TS)

The TS leads to a decrease in skeletal muscle mass and CSA. To investigate the impact of unloading on skeletal muscle contractile performance, we evaluated the gastrocnemius single contractility and fatigue resistance. Our findings indicate that four weeks of unloading resulted in a 44% decrease in maximal gastrocnemius contractile force and a 39% decrease in fatigue resistance (Fig. 5a and b), suggesting degeneration in skeletal muscle function due to the TS.

As expected, HFES with or before the TS led to an increase in gastrocnemius contractility and fatigue resistance in the Pre-H group (38% and 19%, respectively) and Dur-H group (21% and 29%, respectively) (Fig. 5a and b). However, neither group fully restored these parameters to normal levels.

The results of the combined intervention group (H-L) showed an improvement in single contractility (66% increase compared to the TS group) and fatigue resistance (38% increase) that returned to control levels (Fig. 5c and d). The combination of H-L was more effective than other intervention programs in resisting the decline of gastrocnemius muscle mechanical performance, with the maximal single-muscle contraction being increased in the H-L form of electrical stimulation (Fig. 5c). In addition, fatigue resistance showed a similar effect with either H-L or H-H. Therefore, our data suggest that H-L treatment is more effective in resisting the decline of gastrocnemius muscle mechanical performance.

### Skeletal muscle fiber type

**Fig. 6:**
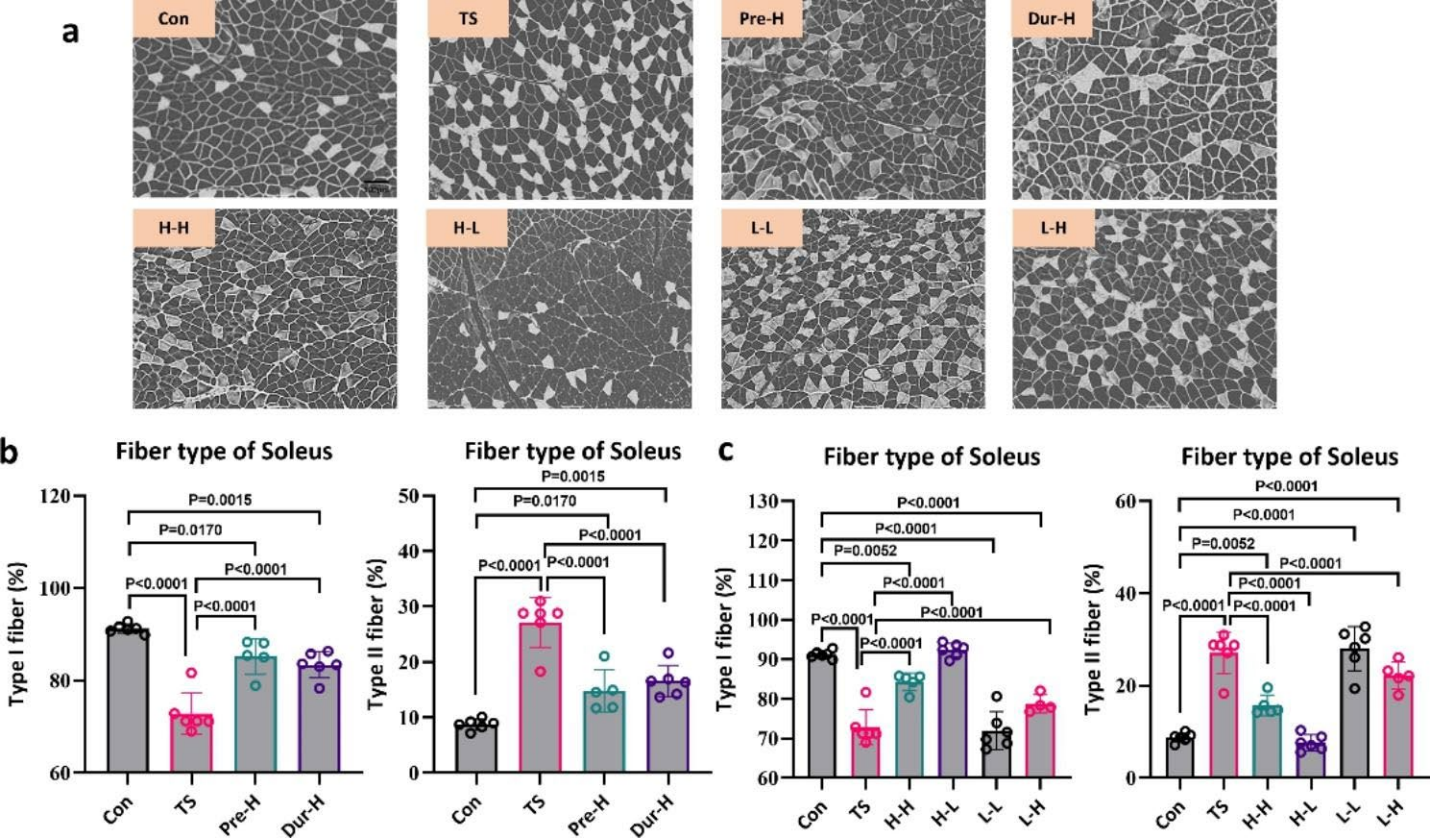
**Electrical stimulation therapy can reverse the changes in soleus muscle fiber types caused by tail suspension** (**a**). The Adenosine triphosphate (ATPase) staining of soleus muscle, the scale bar represents 100 μm. (**b**). The effect of different intervention stages on soleus muscle fiber types. (**c**). The effect of different stimulation schemes on soleus muscle fiber types (Con: control group, TS: Tail suspension group, Pre-H: HFES before TS intervention, Dur-H: HFES during TS, H-H: HFES before and during TS, H-L: HFES before TS plus LFES during TS, L-H: LFES before TS plus HFES during TS, L-L: LFES before and during TS)

**Fig. 7:**
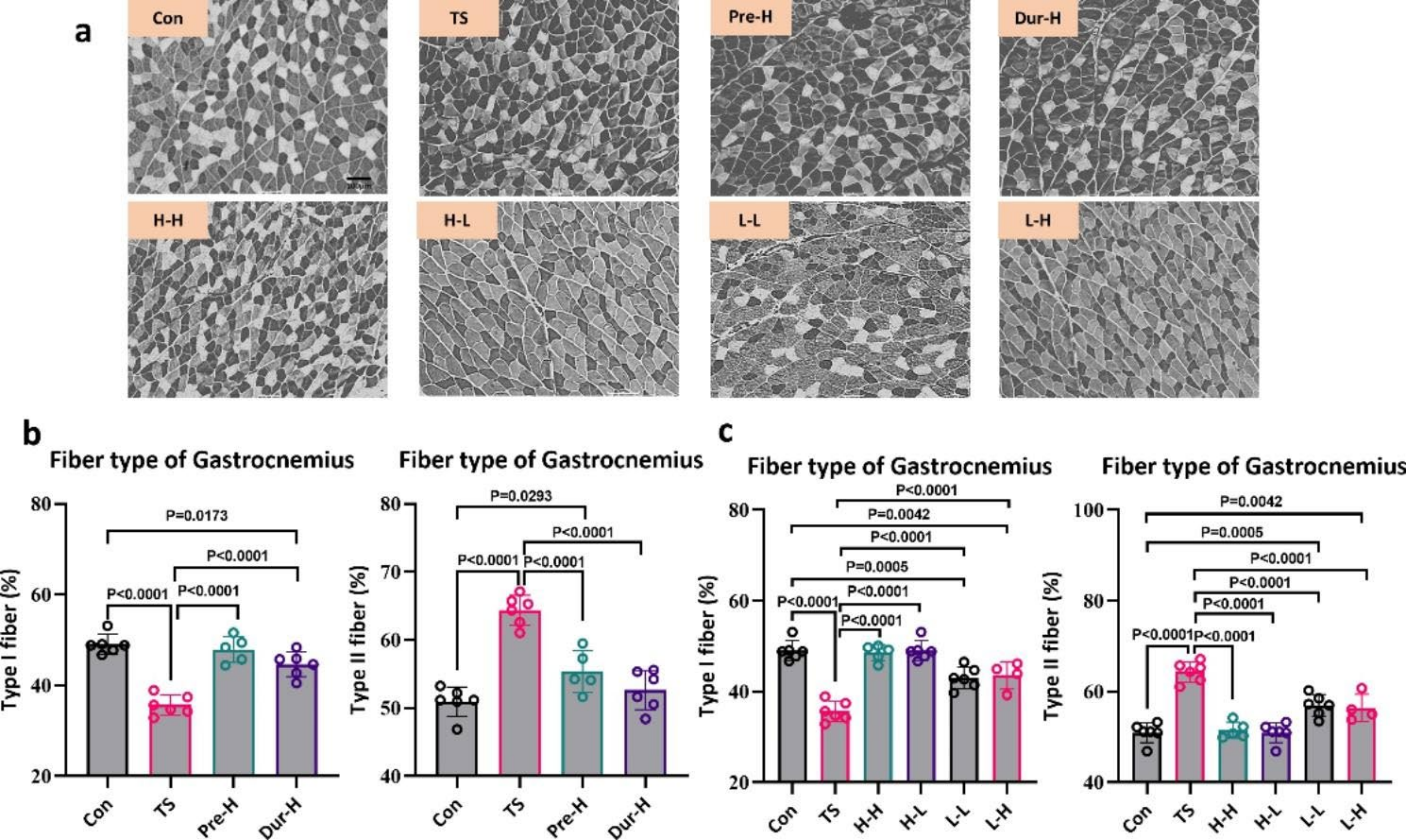
**Electrical stimulation therapy can reverse the changes in gastrocnemius muscle fiber types caused by tail suspension** (**a**). The Adenosine triphosphate (ATPase) staining of gastrocnemius muscle, the scale bar represents 100 μm. (**b**). The effect of different intervention stages on gastrocnemius muscle fiber types. (**c**). The effect of different stimulation schemes on gastrocnemius muscle fiber types (Con: control group, TS: Tail suspension group, Pre-H: HFES before TS intervention, Dur-H: HFES during TS, H-H: HFES before and during TS, H-L: HFES before TS plus LFES during TS, L-H: LFES before TS plus HFES during TS, L-L: LFES before and during TS)

The tail suspension has been shown to cause the conversion of oxidative muscle fibers to glycolytic muscle fibers [43]. We used ATPase staining to examine the effect of unloading on the soleus and gastrocnemius muscle-fiber types. The results showed that four weeks of unloading results in a decrease in the proportion of oxidative muscle fibers from 91 to 73% in the soleus and from 49 to 35% in the gastrocnemius (Figs. 6b and 7b), while the corresponding proportion of glycolytic muscle fibers increased. Both Pre-H and Dur-H stimulation alleviated the fiber type changes caused by tail suspension. Among the combined intervention group, H-L was found to be the most effective in maintaining muscle fiber types, as it improved both the soleus and gastrocnemius muscles, while L-L had no effect on myofiber type in the soleus muscle (Figs. 6b and 7b).

**Fig. 8:**
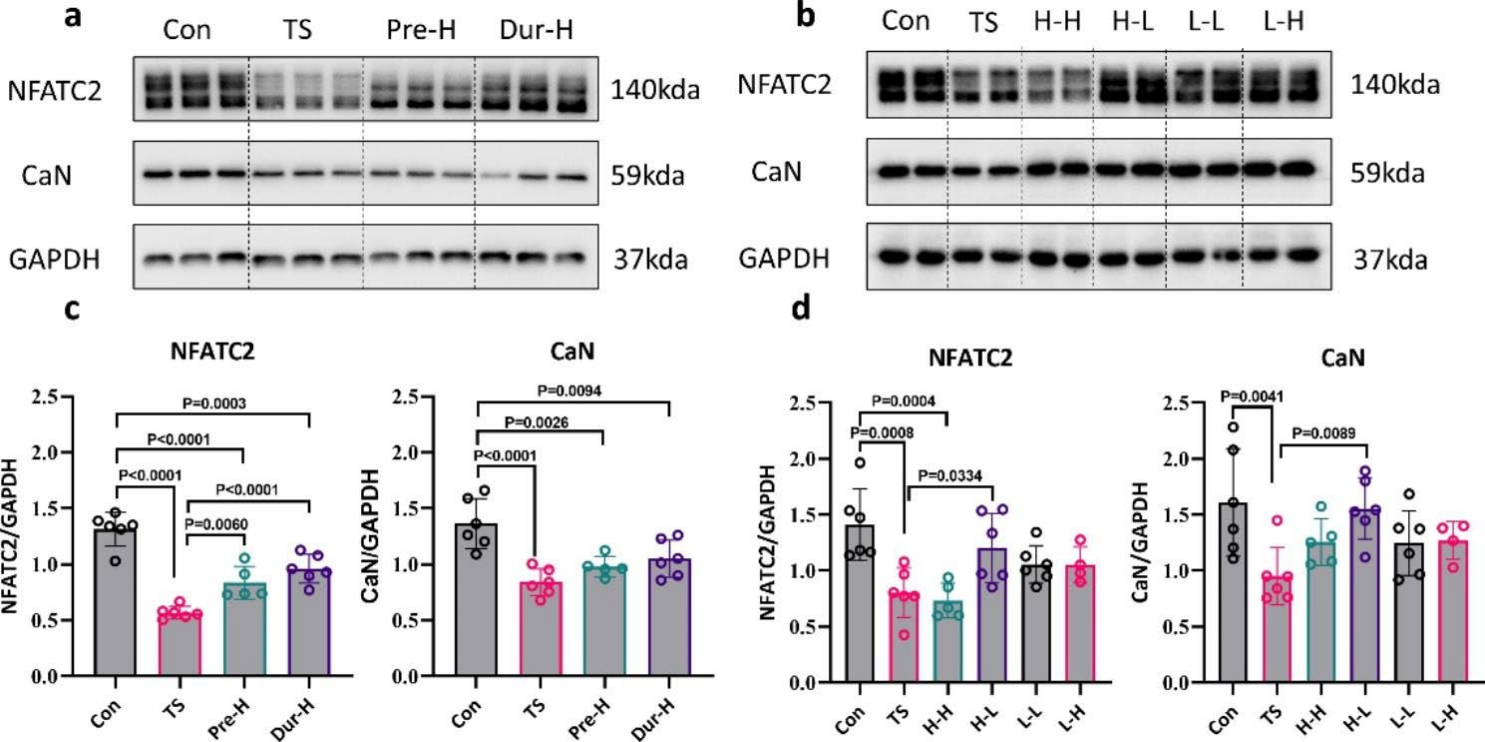
**Electrical stimulation promotes NFATC2 and CaN expression in soleus** (**a**, **b**) Representative Western blotting for NFATC2 and CaN. Tubulin was used as a loading control. (**c**, **d**) The optical density values are normalized to their respective tubulin loading control and the means ± SD are graphed (relative expression) to semi-quantitatively compare the protein levels (Con: control group, TS: Tail suspension group, Pre-H: HFES before TS intervention, Dur-H: HFES during TS, H-H: HFES before and during TS, H-L: HFES before TS plus LFES during TS, L-H: LFES before TS plus HFES during TS, L-L: LFES before and during TS)

NFAT (nuclear factor of activated T cells) is a nerve activity sensor in skeletal muscle that controls myofiber switching [33]. The motor neuron stimulation resulting in a sustained increase in the intracellular calcium-regulatory pool that activates CaN, leading to increased transcription of genes expressed selectively in fatigue-resistant subtypes (Type I) of skeletal myofibers [44]. We found that after 4 weeks of tail suspension, the protein expression of CaN and NFAT in the soleus muscle was significantly lower than that in the Control group, although pre-training with HFES or treatment during unloading was able to up-regulate the protein expression, but not to the levels of the control group (Fig. 8a, c). H-L was found to be the most effective intervention in significantly up-regulating the protein expressions of NFAT and CaN, while H-H, L-L, and L-H had no effect (Fig. 8b, d).

These findings are consistent with the changes in muscle weight, CSA, muscle contractile performance, and muscle fiber type, indicating that H-L is the most efficient method in resisting the decline in skeletal muscle performance caused by tail suspension.

## Discussion

Several studies have shown that unloading leads to a decline in skeletal muscle mass and function [2, 11]. To counteract this, electrical stimulation is employed as a means of promoting the recovery of skeletal muscle mass [23, 45, 46]. However, little research has been done on the use of electrical stimulation to recover skeletal muscle function, such as contractile force and fatigue resistance. The present study aimed to evaluate the therapeutic benefits of various electrical stimulation regimens on skeletal muscle mass and function impacted by muscle disuse.

After 4 weeks of unloading, there was a noticeable decrease in fiber CSA in the gastrocnemius and soleus muscles, along with a shift in the frequency domain to the left. The soleus muscle showed greater atrophy, suggesting that tail suspension has a more pronounced effect on oxidative fibers. After being subjected to different electrical stimulation regimens, the quality of skeletal muscle and the fiber CSA improved, although not fully restoring to the control levels. Our findings indicate that pretreatment with HFES at the early stage of unloading is more effective in preventing atrophy than intervention during unloading. The results of the combined group suggest that LFES treatment during unloading (H-L group) may be more beneficial than HFES before and during unloading(H-H group). The results also indicate that HFES treatment during unloading is not the recommended, however, this study does not provide definitive proof for this conclusion.

Skeletal muscle is categorized based on the predominant type of myosin heavy chain (MHC) gene expressed in motor units. To date, several MHC gene coding proteins, including slow and rapid contractile proteins, have been identified. These MHC proteins determine the inherent contraction rate of skeletal muscle fibers [47]. The contraction speed of skeletal muscle fibers is primarily controlled by motor neurons through the activity mode induced in the fibers, within the range of inherent contraction rate. For example, studies have shown that under low-frequency stimulation, the contraction speed of soleus (Sol) and extensor digitorum longus (Edl) muscles exhibit significant differences due to the inherent differences in their muscle fibers. This internal variation may explain why similar nerve impulse, innervation, or hormones can have varying effects on different types of muscle fibers [48]. Additionally, type I fibers are believed to be particularly sensitive to unloading [49]. Previous studies have demonstrated that after 14 days of hindlimb suspension, 14% of the oxidative muscle fibers protein spectrum is reconfigured from the oxidative muscle fibers to the glycolytic muscle fibers [3]. In the soleus muscle, the proportion of glycolytic muscle fibers increases to more than 40%, whereas under normal conditions, it is less than 15% [50]. Conversely, in the gastrocnemius or anterior tibial muscles, the proportion of oxidative muscle fibers increases and the proportion of glycolytic muscle fibers decreases, indicating that changes in loading state can lead to changes in skeletal muscle fiber type [51]. Our study found that after 4 weeks of suspension, the muscle fiber types of the soleus and gastrocnemius muscles changed compared with the control group. The proportion of oxidative muscle fibers in the soleus and gastrocnemius muscles decreased by 21% and 29%, respectively.

The composition of skeletal muscle fiber is influenced by various loading states and different forms of contraction [52–54]. Tonic motor activity at 10–15 Hz, which are typical of slow-twitch muscle fibers, result in a sustained increase in Ca^2+^ concentration ranged from 100 to 300 nm [55], which activates the expression of calcineurin and NFAT protein, leading to increased capillary density and mitochondrial activity and a gradual transformation of fast-twitch muscle fibers into slow-twitch muscle fibers [56]. Our study found that unloading caused a significant decrease in the content of oxidative muscle fibers in soleus muscle, and that LFES was more effective in treating this issue compared to HFES. This may be since LFES mimics the slow-twitch muscle fiber-dominant contraction of soleus muscle, preserving the expression of CaN and NFAT proteins and the morphological and histological characteristics of oxidized muscle fibers. While HFES may alleviate the changes in muscle fiber types during unloading, it did not fully restore it to the control level. However, if LFES treatment is used before and during unloading, the effect will be greatly reduced, which further demonstrates the effectiveness of LFES treatment during unloading after HFES pretreatment.

Studies have shown that after undergoing tail suspension and reloading, mice exhibit a significant decrease in their skeletal muscle function, suggesting that the skeletal muscle strength of mice does not return to normal levels naturally [11, 36, 54]. The decrease in muscle strength may be due to the reduction of contractile tissue in the gastrocnemius muscle, but it is also possible that the gastrocnemius muscle’s reduced strength is a result of a decline in fluid levels in the hind limbs caused by the unloading process. This decline in fluid levels may affect the function of fast-contracting muscles, which are known to be more sensitive to changes in hydration [57, 58]. Thus, the decline in skeletal muscle function could be a result of the decrease quality of gastrocnemius contraction tissue and fluid imbalances.

Consideration must also be given to the fact that a 4-week period of unloading can result in skeletal muscle damage and impairments in neuromuscular control [59]. The decrease in muscle strength during unloading was found to be significantly greater than the loss of muscle mass. The gastrocnemius muscle mass at the end of unloading was no different from that of the control group. H&E staining results showed no evidence of skeletal muscle damage and repair, indicating that tail suspension does not cause skeletal muscle damage, but we cannot rule out the possibility of nerve damage or dysfunction affecting skeletal muscle function.

Although tail suspension leads to skeletal muscle dysfunction, single contractility and fatigue resistance were found to be significantly lower than normal levels. This study discovered that electrical stimulation can partially alleviate the decline in skeletal muscle function. This may be due to electrical stimulation maintaining involuntary contraction activity, enhancing the quality of contractile tissue, and preserving fiber type, or the ability of electrical stimulation to simulate the nervous system in controlling muscle contraction. Ultimately, the result is increased contractile activity of skeletal muscle and the preservation of its function.

## Conclusion

Present study highlights the rapid loss of skeletal muscle mass and a significant reduction in fiber CSA because of a 4-week unloading intervention. The fiber type also changed, as observed in other studies, resulting in varying degrees of decline in the maximal single contractile force and fatigue resistance of the gastrocnemius muscle. We found that pretreatment with HFES followed by continuous LFES during unloading can preserve the contractile function of skeletal muscle by counteracting the pathological effects of muscle atrophy, fiber area reduction, and fiber type change caused by tail suspension. However, further studies are required to investigate the effects of different frequency electrical stimulation on the different types of skeletal muscle fibers at different loading stages, as well as the underlying regulatory factors.

Our study has several limitations that should be noted. Firstly, female animals can introduce additional variables due to the impact of their physiological period, which is why we focused only on male animals. Secondly, while we found that a combination of HFES pretreatment followed by LFES during tail suspension can prevent degradation of the mechanical properties of the gastrocnemius muscle, further exploration is needed to determine the best method of applying these findings in practice. Finally, while tail suspension was used to create a skeletal muscle atrophy model, it is not directly comparable to the muscle atrophy and complications that result from aging, long-term bed rest, or other conditions. However, this study still provides valuable insights for special populations, such as astronauts.

## Data Availability

The datasets used and/or analyzed in this study are available upon reasonable request from the corresponding author.

## Abbreviations

LFES: Low-frequency electrical stimulation
HFES: High-frequency electrical stimulation
SD: Sprague-Dawley
TS: Tail suspension
Pre-H: HFES pretreatment and then tail suspension
Dur-H: HFES training during tail suspension
H-H: HFES pretreatment then HFES during tail suspension
H-L: HFES pretreatment then LFES during tail suspension
L-H: LFES pretreatment then HFES during tail suspension
L-L: LFES pretreatment then LFES during tail suspension
CSA: Cross section area
FES: Functional electrical stimulation
NFAT: Nuclear factor of activated T cell
CaN: Calcineurin
ATPase: Adenosine triphosphatase
H&E: Hematoxylin-eosin
MHC: Myosin heavy chain
