# Correction: An electrical stimulation intervention protocol to prevent disuse atrophy and muscle strength decline: an experimental study in rat

**DOI:** 10.1186/s12984-026-02117-0

**Published:** 2026-08-08

**Authors:** Haiwang Shi, Fan Li, Fulong Zhang, Xiaobei Wei, Chengyi Liu, Rui Duan

**Affiliations:** Lab of Regenerative Medicine in Sports Science, School of Physical Education and Sports Science, South China Normal University, Guangzhou, China

**Correction: Journal of NeuroEngineering and Rehabilitation (2023) 20:84** 10.1186/s12984-023-01208-6

Following publication, concerns were raised regarding the presentation of images in Fig. 4a and d of this article [1], including overlap between figure panels and corresponding images in a related publication by the same authors [2]. The authors have clarified that the affected panels were representative images from the same broader experimental work. To address the concerns, the affected figures have been replaced with replicate images.

The correction does not affect the results or conclusions of the article.
